# Synthesis and Biological Evaluation of a Lipophilic Vorinostat Prodrug

**DOI:** 10.1002/cbdv.71726

**Published:** 2026-09-27

**Authors:** Navneet Thakur, Neha Bhardwaj, Sudesh Kumar Yadav, Ankit Saneja

**Affiliations:** ^1^ Formulation Laboratory, Dietetics and Nutrition Technology Division CSIR‐Institute of Himalayan Bioresource Technology Palampur Himachal Pradesh India; ^2^ Academy of Scientific and Innovative Research (AcSIR) Ghaziabad India; ^3^ CSIR‐Institute of Himalayan Bioresource Technology Palampur Himachal Pradesh India

**Keywords:** anticancer, apoptosis, lipid‐drug conjugation, prodrug, reactive oxygen species, vorinostat

## Abstract

Vorinostat, a hydroxamic acid‐based histone deacetylase (HDAC) inhibitor approved for cutaneous T‐cell lymphoma, exhibits broad‐spectrum anticancer activity. However, its clinical application is limited by poor permeability and rapid metabolism, which result in a short half‐life and consequently poor therapeutic efficacy. To address these constraints, we synthesized a series of lipid‐vorinostat conjugates (3a‐d) through acylation of vorinostat with short‐chain fatty acid chloride derivatives (2a‐d). The chemical structures and purity of the conjugates (3a‐d) were confirmed using NMR, HRMS, and HPLC analyses. Among these derivatives, compound 3d (3,5,5‐Trimethylhexanoyl‐Vorinostat) demonstrated improved cytotoxicity against A549 and 4T1 carcinoma cells. Mechanistic insight revealed that the compound 3d induced higher levels of caspase‐3/7 activation, reactive oxygen species (ROS) generation, and mitochondrial membrane potential (MMP) loss compared with vorinostat, suggesting enhanced apoptosis. The cell scratch assay demonstrated that 3d markedly inhibited cell migration as compared with vorinostat. Furthermore, in silico ADMET profiling predicted enhanced membrane permeability of the compound 3d as compared to vorinostat. The in vitro plasma stability revealed time‐dependent hydrolysis of compound 3d to release active vorinostat, supporting its prodrug behavior. Collectively, these findings suggest lipid conjugation as a promising prodrug strategy to modulate the biological and physicochemical properties of vorinostat.

## Graphical abstract

A series of lipid‐vorinostat conjugates (3a‐d) through acylation with short‐chain fatty acid chloride derivatives were synthesized. The compound 3d demonstrated improved cytotoxicity against A549 and 4T1 carcinoma cells. Mechanistic insight revealed that the prodrug 3d induced higher levels of caspase‐3/7 activation, reactive oxygen species (ROS), and mitochondrial membrane potential (MMP) loss.

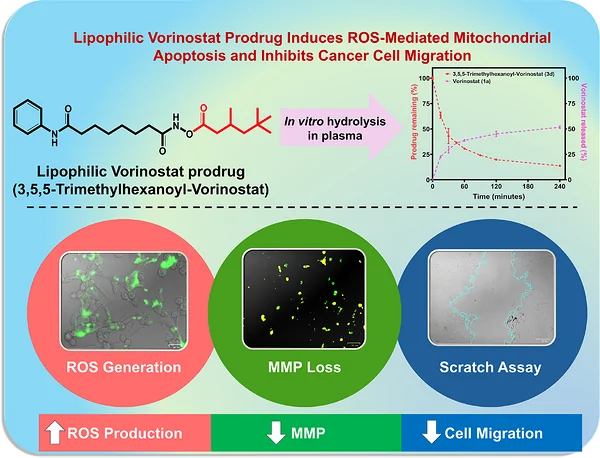

## 1 Introduction

Cancer remains one of the leading causes of mortality worldwide, with a growing burden on healthcare systems. According to recent reports, approximately 20 million new cancer cases and 9.7 million cancer‐related deaths were recorded worldwide in 2022, emphasizing the urgent need for effective treatment strategies [1]. In addition, the increasing incidence of early‐onset cancers among adults younger than 50 years further highlights the growing global burden of cancer [2]. By 2050, the global incidence of cancer is projected to rise to 35 million new cases annually [1]. Despite major advances in cancer therapeutics, the clinical management of many cancers remains challenging due to issues such as poor drug bioavailability and systemic toxicity [3]. These limitations are largely attributed to suboptimal physicochemical and pharmacokinetic profiles, and compound‐specific safety concerns, often resulting in inconsistent intratumor drug concentrations [4].

Vorinostat (suberoylanilide hydroxamic acid, SAHA) is a first‐in‐class histone deacetylase (HDAC) inhibitor for the treatment of cutaneous T‐cell lymphoma, approved by the FDA [5]. It functions by inhibiting class I and II HDACs through chelation of the Zn^2^^+^ ion at the catalytic site of the enzyme, thereby promoting hyperacetylation of histones and transcription factors, leading to cell cycle arrest and apoptosis [6]. Vorinostat has shown promising therapeutic activity against a range of malignancies, including solid tumors and glioblastoma [7, 8, 9]. However, its clinical efficacy is limited by low membrane permeability, and rapid metabolic degradation, which compromises its pharmacokinetic properties [10]. Vorinostat has a short plasma half‐life of approximately two hours when administered orally [10]. Collectively, these limitations lead to insufficient drug accumulation at tumor sites and reduced therapeutic outcomes

Hence, to modulate the permeability and efficacy constraints of vorinostat, chemical modification strategies such as lipid‐drug conjugation (LDC) can be explored as a prodrug strategy. LDC involves the covalent attachment of lipid moieties to pharmacologically active agents to form prodrug, primarily aiming to enhance lipophilicity, membrane permeability, and promotes preferential tumor accumulation. Although this structural modification may reduce intrinsic aqueous solubility, it strategically modulates the overall physicochemical and pharmacokinetic profile to improve efficacy and tumor targeting efficiency [11, 12, 13, 14, 15]. Prodrugs are pharmacologically inactive derivatives of parent compounds that are designed to undergo bioconversion, releasing the active drug through inherent structural lability [16, 17]. This conversion is typically mediated by enzymatic or physicochemical processes, thereby reinstating therapeutic activity at the target site. The efficiency of LDC depends on the chemical compatibility between the drug and the lipid moiety, as well as the nature of the linker and release mechanism. The common approaches include fatty acid‐drug conjugation, glyceride conjugation, steroid conjugation, and phospholipid‐based conjugation [15, 18].

In this study, vorinostat was chemically modified *via* acylation with different acid chlorides derived from short‐chain fatty acids to synthesize lipophilic vorinostat conjugates bearing O‐acyl hydroxamate linkages. The rationale for this design was to exploit the O‐acyl hydroxamate linkage as a cleavable modification while allowing the structure of the short‐chain fatty acid moiety to potentially modulate the physicochemical and hydrolytic properties of the resulting prodrugs. O‐Acylation of the hydroxamate group masks the parent hydroxamate functionality and generates a linkage that may undergo hydrolytic and/or enzyme‐mediated cleavage under physiological conditions, thereby regenerating the active parent hydroxamate [9]. The resulting conjugates were thoroughly characterized using NMR, HRMS, and HPLC. Among the synthesized conjugates, vorinostat prodrug (3d), functionalized with 3,5,5‐Trimethylhexanoyl group, demonstrated superior cytotoxic activity against human lung adenocarcinoma (A549) and murine mammary carcinoma (4T1) cell lines. This prodrug was further evaluated for its anticancer activity through assays including caspase‐3/7 activation, reactive oxygen species (ROS) generation, mitochondrial membrane potential (MMP) loss, and cell migration inhibition via scratch assay. In silico ADMET profiling of the synthesized conjugates (3a‐d) was performed to assess their pharmacokinetic properties and drug likeliness. Moreover, the in vitro plasma stability of prodrug 3d was performed to assess its hydrolysis and conversion to active vorinostat.

## 2 Results and Discussion

### 2.1 Synthesis and Characterization of Lipid‐Vorinostat Conjugates

Lipid‐Vorinostat conjugates were synthesized via acylation of vorinostat with acid chloride derivatives of short‐chain fatty acids, forming O‐acyl hydroxamate linkages (Scheme 1). Although N‐acylation is possible, O‐acylation is expected to be favored under the reaction conditions due to the reduced nucleophilicity of the resonance‐stabilized hydroxamic acid nitrogen. The successful synthesis of Isovaleryl‐Vorinostat conjugate (3a), Nonanoyl‐Vorinostat (3b), Heptanoyl‐Vorinostat (3c), and 3,5,5‐Trimethylhexanoyl‐Vorinostat (3d) was confirmed by NMR, HRMS, and HPLC analyses (Table 1 and Figures S1–S15).

**SCHEME 1:**
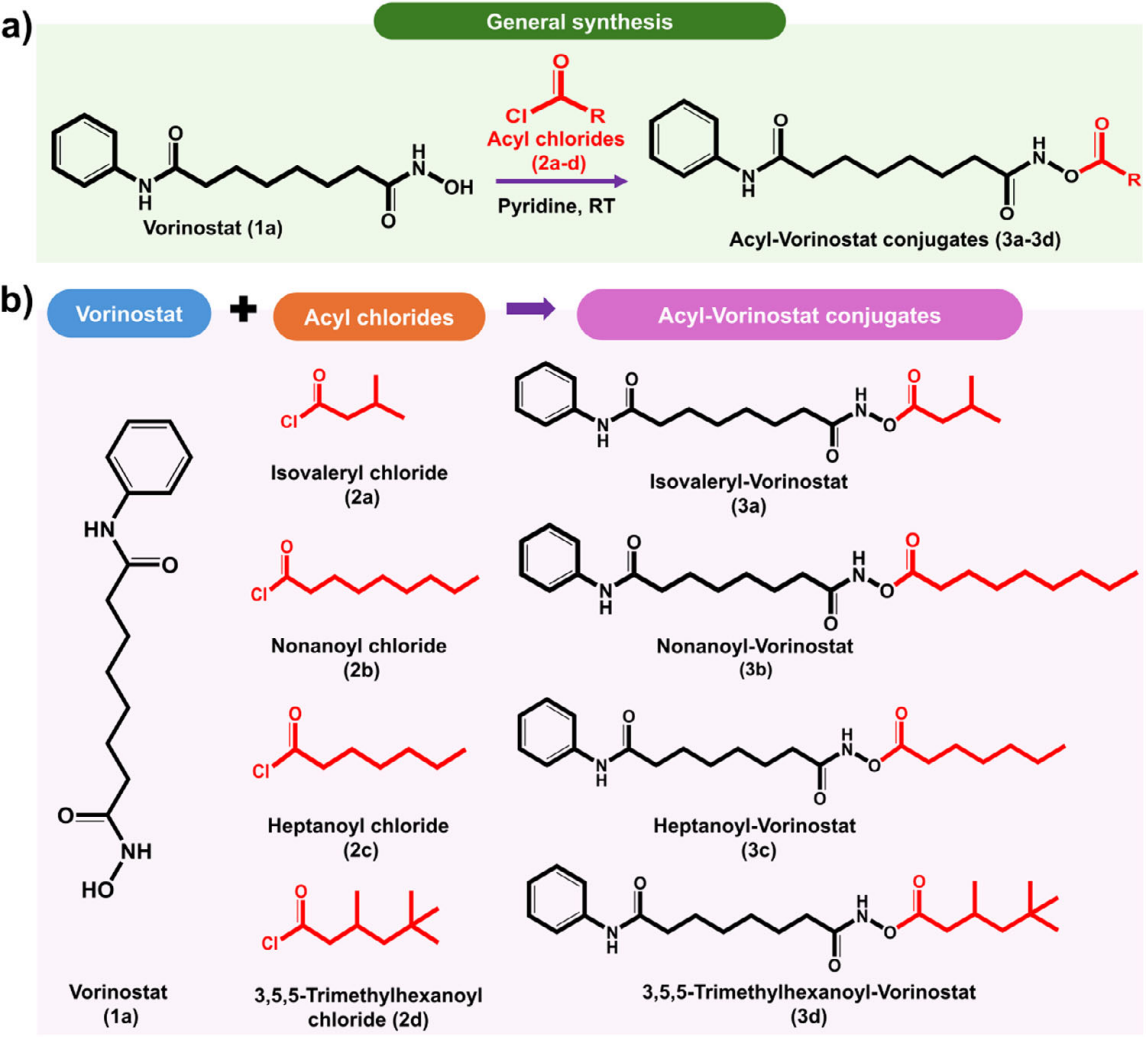
General synthesis and structure of Acyl‐Vorinostat conjugates. a) General reaction scheme illustrating the synthesis of Acyl‐Vorinostat derivatives (3a‐d), via the reaction of vorinostat (1a) with various acyl chlorides in the presence of pyridine for 36 hours at room temperature. b) Structures of the specific acyl chlorides (2a‐d) used and the corresponding vorinostat conjugate synthesized: Isovaleryl‐Vorinostat (3a), Nonanoyl‐Vorinostat (3b), Heptanoyl‐Vorinostat (3c), and 3,5,5‐Trimethylhexanoyl‐Vorinostat (3d).

**TABLE 1:** Characterization of vorinostat (1a) and its conjugates (3a‐d) by HRMS and HPLC Analysis.

S. no.	Compounds	Molecular formula	Mass [M+H]^+^ (g/mol)		Retention time (minutes)	Purity (%)	Yield (%)
			Expected	Observed			
1	Vorinostat (**1a**)	C_14_H_20_N_2_O_3_	265.1547	265.1552	3.86	99.89	na
2	Isovaleryl‐Vorinostat (**3a**)	C_19_H_28_N_2_O_4_	349.2122	349.2123	4.90	98.43	53
3	Nonanoyl‐Vorinostat (**3b**)	C_23_H_36_N_2_O_4_	405.2748	405.2750	10.04	95.05	65
4	Heptanoyl‐Vorinostat (**3c**)	C_21_H_32_N_2_O_4_	377.2435	377.2439	6.54	96.13	58
5	3,5,5‐Trimethylhexanoyl‐Vorinostat (**3d**)	C_23_H_36_N_2_O_4_	405.2748	405.2749	8.21	99.20	57

### 2.2 Cytotoxicity Evaluation of Vorinostat Conjugates

The cytotoxic effects of vorinostat (1a) and its lipid conjugates (3a‐d) were evaluated against A549 and 4T1 cells. While the primary objective of the LDC strategy was to modulate the pharmacokinetic profile of vorinostat (1a), in vitro cytotoxicity assays against A549 and 4T1 cells were employed as a functional triage. This approach provides biologically relevant efficacy evaluation of synthesized vorinostat conjugates. Among the synthesized conjugates, compound 3d exhibited superior cytotoxicity with significantly lower IC_50_ values in A549 (5.77 ± 1.12 µM *vs* 9.71 ± 1.13 µM) and 4T1 cells (4.29 ± 0.28 µM *vs* 9.89 ± 0.35 µM) as compared to native vorinostat (Table 2 and Figure S16). The observed IC_50_ values for vorinostat align well in accordance with the literature [19, 20]. The compound 3d demonstrated greater cytotoxic efficacy compared with the other synthesized vorinostat conjugates, likely owing to its optimal balance of physicochemical properties rather than increased lipophilicity alone. Further biocompatibility studies were conducted on murine macrophage (RAW 264.7) and fibroblast (L929) cells to assess the safety profile of the potential compound 3d and vorinostat (1a) (Figure S17). Interestingly, both compounds demonstrated minimal cytotoxicity toward normal cells, maintaining more than 80% cell viability at the highest tested concentration (40 µM), its selectivity index (SI) was estimated to be > 6.5 and > 9.0 for the A549 and 4T1 cell lines, respectively, indicating preferential cytotoxicity toward cancer cells.

**TABLE 2:** IC_50_ values (µM) of vorinostat and its conjugates (3a‐d) against A549 and 4T1 cell lines after 48 hours.

S no.	Compounds	A549	4T1
1	Vorinostat (**1a**)	9.719 ± 1.13	9.897 ± 0.35
2	Isovaleryl‐Vorinostat (**3a**)	17.582 ± 1.72	18.971 ± 0.91
3	Nonanoyl‐Vorinostat (**3b**)	19.769 ± 0.95	>20
4	Heptanoyl‐Vorinostat (**3c**)	>20	17.979 ± 1.10
5	3,5,5‐Trimethylhexanoyl‐Vorinostat (**3d**)	5.772 ± 1.12^a^	4.298 ± 0.28^a^

^a^ p indicates signicant difference compared to 1a, with *p* < 0.05, (n = 3)

### 2.3 Caspase 3/7 Activity

**FIGURE 1:**
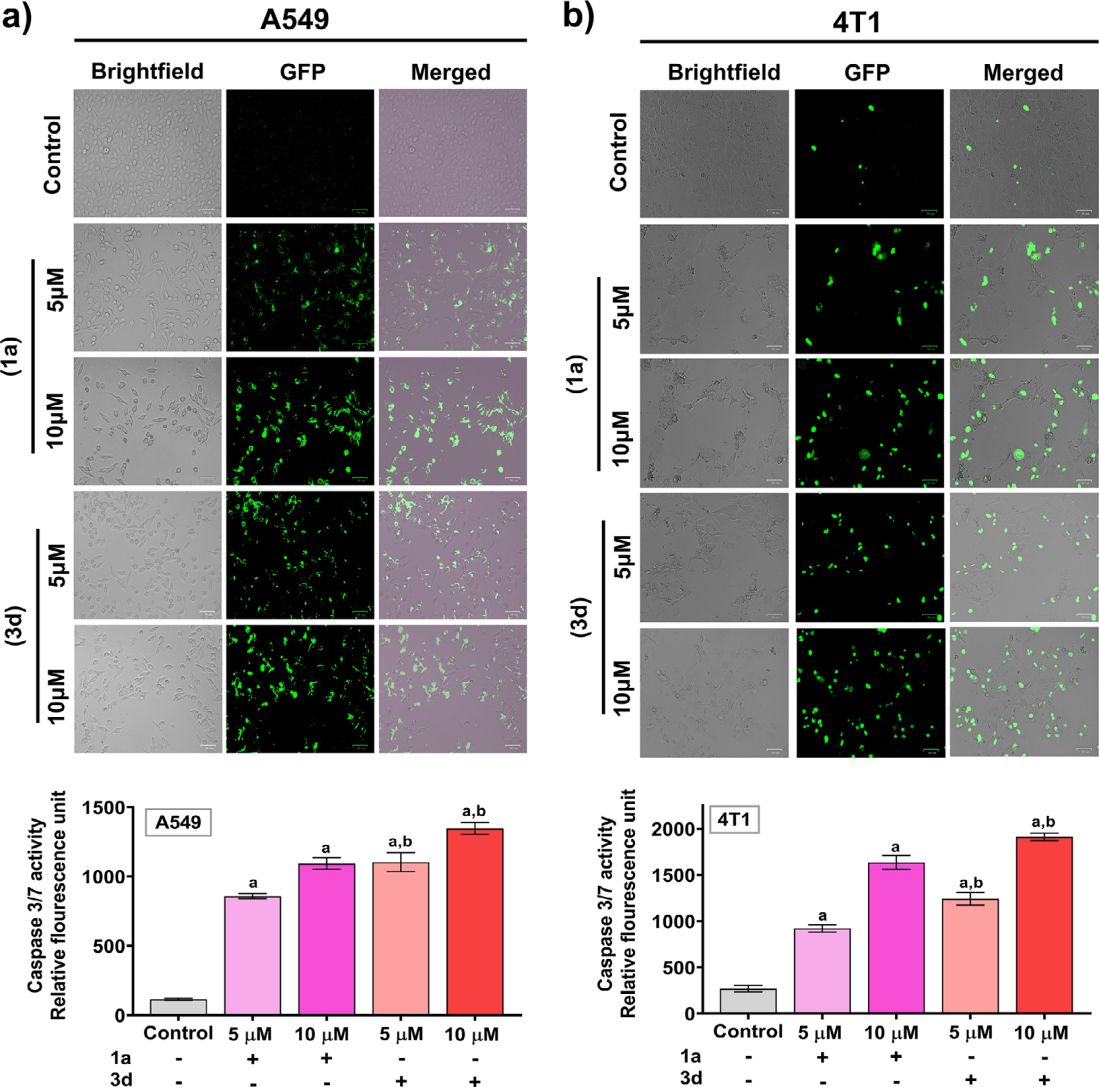
Evaluation of caspase 3/7 activity in A549 and 4T1 cells after treatment with vorinostat (1a) and its conjugate (3d). (a) Representative fluorescence images and quantification of caspase 3/7 activity in A549 cells following 24‐hour treatment with 5 µM and 10 µM of 1a and 3d. (b) Caspase 3/7 activation in 4T1 cells under similar treatment conditions. Quantitative data are shown as relative fluorescence units (RFU). The data were expressed as mean ± SD (n = 3). a: p < 0.05 vs. control group; b: p < 0.05 vs. 1a‐treated group at the same concentration. Treatment with 3d induced significantly enhanced caspase 3/7 activity in comparison to 1a.

Caspases, a family of cysteine proteases, play a crucial role in the initiation and execution of apoptosis [21]. They are broadly classified into initiator and effector caspases. Initially produced as inactive procaspases, these enzymes are activated by apoptotic stimuli. Among the effector caspases, caspase‐3 and caspase‐7 serve as key executioners; caspase‐3 primarily drives DNA fragmentation and morphological changes during apoptosis, while caspase‐7, though similar in function, is more directly linked to the loss of cell viability [22]. In both A549 and 4T1 cancer cell lines, treatment with vorinostat (1a) and synthesized conjugate 3d demonstrated significantly enhanced caspase‐3/7 activity in comparison to untreated controls. The fold change was calculated relative to the untreated control group. In A549 cells, compound 3d showed a stronger effect with (9.7 vs 7.6 ‐fold) and (11.9 vs 9.6 ‐fold) increases in caspase‐3/7 activity than vorinostat at 5 µM and 10 µM, respectively, relative to untreated control (Figure 1). Similarly, in 4T1 cells, the compound 3d compared to 1a demonstrated (4.6 vs 3.4 ‐fold) and (7.1 vs 6.0 ‐fold) increases in caspase‐3/7 activity at 5 µM and 10 µM, respectively, relative to the untreated control group. This pronounced elevation in caspase‐3/7 activity by compound 3d indicates a stronger pro‐apoptotic effect as compared to native vorinostat.

### 2.4 Vorinostat Prodrug (3d) Induces ROS‐Mediated Oxidative Stress in Cancer Cells

**FIGURE 2:**
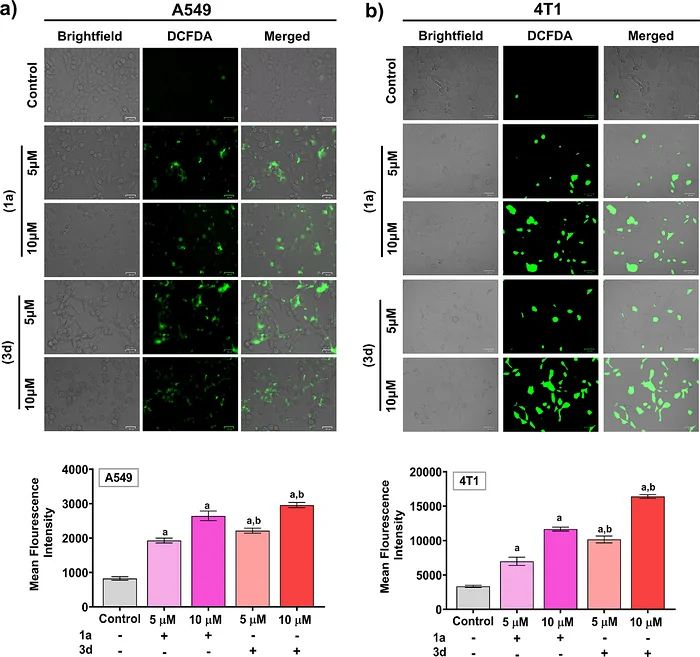
Intracellular ROS accumulation induced by vorinostat (1a) and its conjugate (3d) in cancer cells. (a) Representative fluorescence images (brightfield, DCFDA fluorescence, and merged) and quantification of ROS levels in A549 cells after treatment with 5 µM and 10 µM concentrations of 1a and 3d for 24 hours. (b) Analysis in 4T1 cells under the same treatment conditions. ROS generation was detected using the DCFDA fluorescent probe, where green fluorescence indicates ROS generation. Quantification was performed using a microplate reader and expressed as mean fluorescence intensity. All data are presented as mean ± SD (n = 3). a: p < 0.05 vs. control group; b: p < 0.05 vs. 1a‐treated group at the same concentration. Treatment with compound 3d led to a significantly higher ROS accumulation in both A549 and 4T1 cells compared to 1a.

ROS exhibits dual role in cellular physiology, favoring normal functions at low levels but triggering apoptosis when excessively accumulated, particularly in cancer cells [22]. Most anticancer agents exert their effects through generation of ROS. In this study, DCFDA assay was utilized to assess the intracellular ROS levels, a nonfluorescent probe converted into a fluorescent compound upon oxidation by ROS [23]. Both vorinostat (1a) and its synthesized conjugate (3d) significantly increased ROS levels in A549 and 4T1 cancer cells at 5 and 10 µM concentrations. In A549 cells, the compound 3d led to marked increase in fluorescence with 2.7 and 3.6 ‐fold at 5 and 10 µM, respectively, compared to the control. Similarly, in 4T1 cells, compound 3d enhanced ROS levels by 3.0 and 4.9 ‐fold at aforementioned concentrations (Figure 2). These results clearly demonstrated that lipid modification of vorinostat enhances its ability to induce ROS‐mediated oxidative stress, contributing to its enhanced anticancer efficacy.

### 2.5 Mitochondrial Membrane Potential Loss Induced by Vorinostat Conjugate (3d)

**FIGURE 3:**
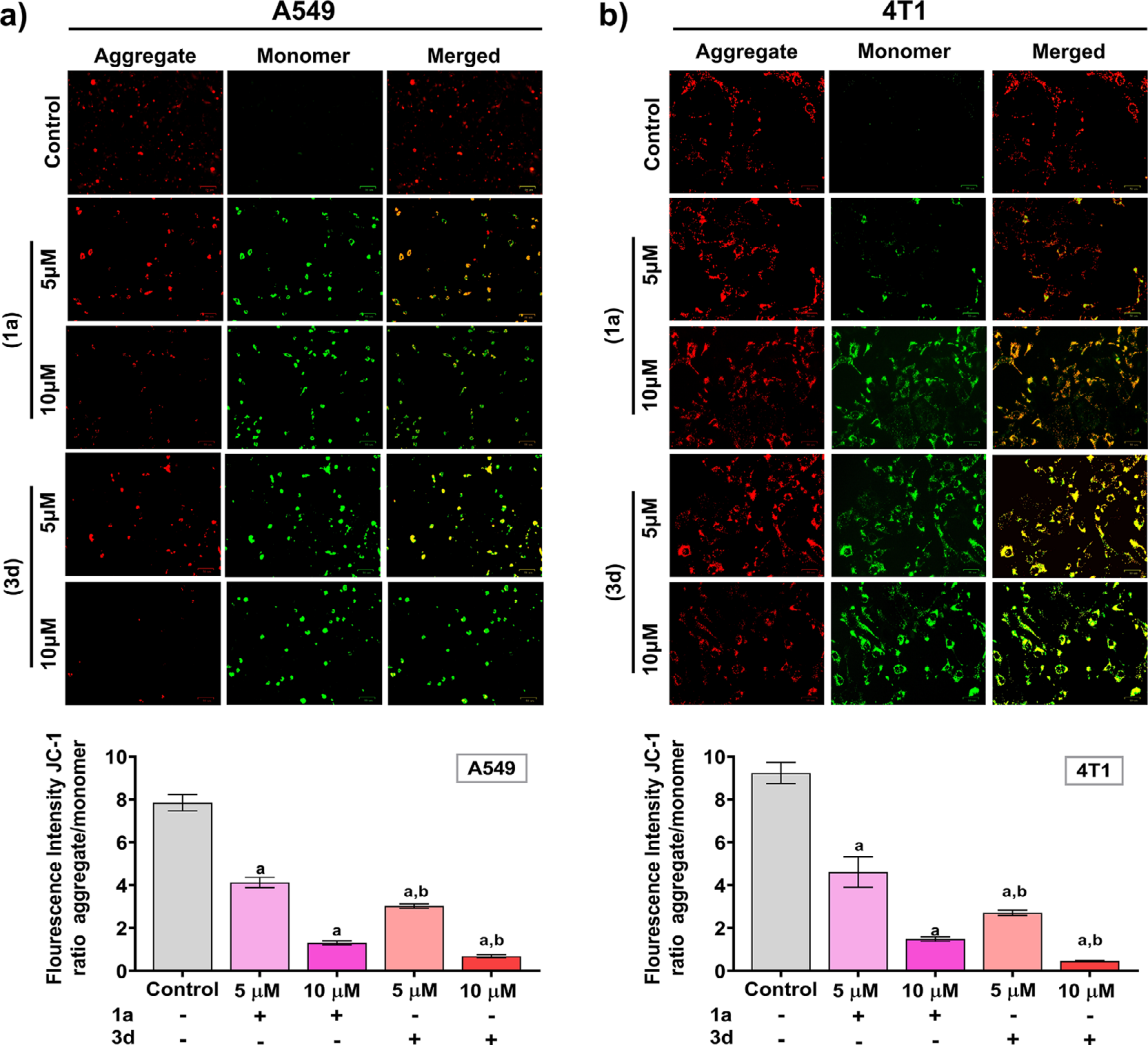
Effect of vorinostat (1a) and its conjugate (3d) on mitochondrial membrane potential (MMP) in A549 and 4T1 cells. (a) Fluorescence microscopy images and quantification of JC‐1 staining‐ A549 cells treated with 5 µM and 10 µM of 1a and 3d for 24 hours. (b) Analysis in 4T1 cells under the same treatment conditions. JC‐1 dye aggregates emit red fluorescence, while monomers emit green fluorescence. The merged images show the transition in mitochondrial potential. Quantitative analysis expressed as the red/green fluorescence intensity ratio (JC‐1 aggregate/monomer), indicating mitochondrial health. The data were presented as mean ± SD (n = 3). a: p < 0.05 vs. control group; b: p < 0.05 vs. 1a‐treated group at the same concentration. Treatment with compound 3d demonstrated a more distinct decrease in the JC‐1 ratio.

MMP is a vital indicator of mitochondrial function and typically elevated in carcinoma cells [24]. Excessive ROS accumulation, often triggered by anticancer agents, can compromise mitochondrial integrity, leading to MMP depolarization and activation of intrinsic apoptotic pathways [25]. To assess MMP disruption, JC‐1 dye was used. In healthy cells, JC‐1 forms red‐fluorescent J‐aggregates, whereas in apoptotic cells with compromised MMP, it remains in its green‐fluorescent monomeric form. The red‐to‐green fluorescence ratio provides a quantitative measure of MMP integrity. In A549 cells, untreated controls exhibited a high JC‐1 ratio (∼7.85), reflecting intact MMP. Treatment with vorinostat (1a) reduced this ratio to ∼4.12 and ∼1.3 at 5 µM and 10 µM, respectively. The lipid‐Vorinostat conjugate (3d) caused a more significant loss of MMP, with JC‐1 ratios dropping to ∼3.02 and ∼0.68 at the same concentrations. A similar pattern was observed in 4T1 cells. The control group showed a JC‐1 ratio of ∼9.23, while 1a‐treated cells displayed ratios of ∼4.6 and ∼1.48. Compound 3d further reduced the MMP with JC‐1 ratios of ∼2.71 and ∼0.46 at 5 and 10 µM, respectively (Figure 3). These findings confirm that 3d induces more pronounced mitochondrial depolarization than vorinostat, supporting its enhanced pro‐apoptotic and anticancer activity.

### 2.6 Inhibitory Effect of Prodrug 3d on Cell Migration

**FIGURE 4:**
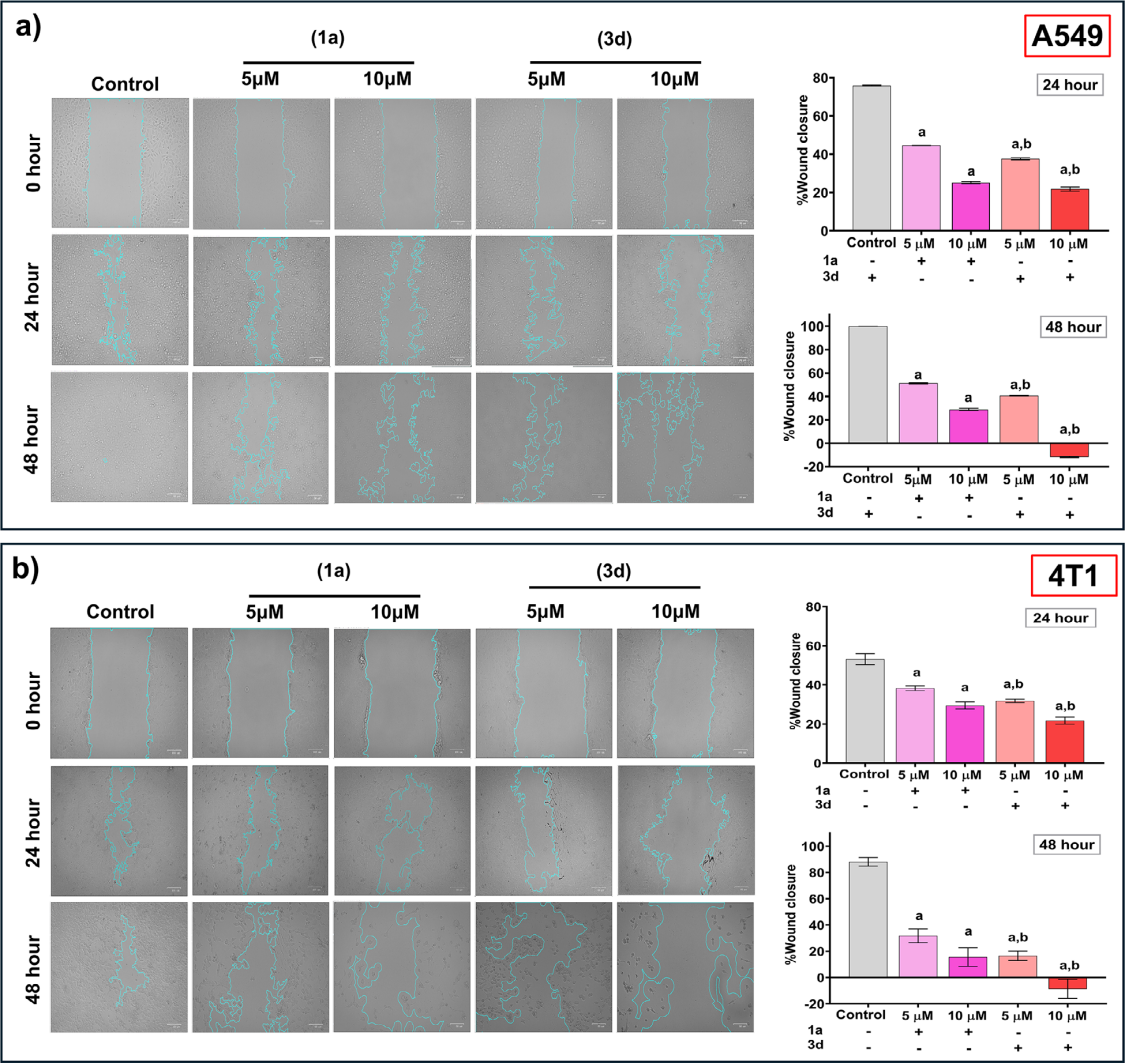
Scratch wound assay to evaluate anti‐migratory and anti‐proliferative effects of vorinostat (1a) and its prodrug (3d) on cancer cells. (a) Representative images and quantitative analysis of wound closure in A549 cells at 0, 24 and 48 ‐hours post‐treatment with 5 µM and 10 µM concentrations of 1a and 3d. (b) Representative images and quantification of wound closure in 4T1 cells at similar time points and concentrations. Wound closure was measured using ImageJ and expressed as % wound closure relative to 0 hour. All data were presented as mean ± SD (n = 3). a: p < 0.05 vs. control group; b: p < 0.05 vs. 1a‐treated group at the same concentration (5 µM or 10 µM). Treatment with compound 3d showed significantly reduced wound closure in both cell lines compared to vorinostat (1a).

Metastasis, a defining feature of malignant tumors, involves the uncontrolled proliferation and migration of cancer cells [26]. To investigate the anti‐proliferative and anti‐migratory potential of the lipid‐vorinostat conjugate (3d), an in vitro scratch assay (wound healing) was performed using A549 and 4T1 cell lines. In A549 cells, untreated controls showed a wound closure of 75.84 ± 0.47% at 24 hours. Treatment with vorinostat (1a) at 10 µM reduced wound closure to 25.12 ± 1.00%, while compound 3d further decreased it to 21.78 ± 1.85%, indicating superior inhibition of cell migration and proliferation. After 48 hours, the difference became more pronounced, with compound 3d leading to a negative wound closure of ‐11.83 ± 0.38%, indicating wound expansion due to cell death, whereas 1a showed 28.80 ± 0.92%. In contrast, untreated cells nearly regained full confluency (99.86 ± 0.01%) (Figure 4a). Similarly, in 4T1 cells, compound 3d consistently outperformed 1a. At 24 hours, wound closure was 53.18 ± 2.79% (control), 29.48 ± 1.81% (1a), and 21.76 ± 1.78% (3d) at 10 µM concentration. At 48 hours, untreated cells showed 88.11 ± 3.27% wound closure, whereas 3d markedly inhibited the migration with ‐8.59 ± 7.27 % at 10 µM, compared to 1a with 15.64 ± 7.14 % wound closure (Figure 4b). These results confirm that lipid conjugation of vorinostat enhances anti‐migratory effects, with compound 3d demonstrating superior anticancer potential.

### 2.7 In Silico ADMET Profile of Vorinostat and Its Conjugates

**FIGURE 5:**
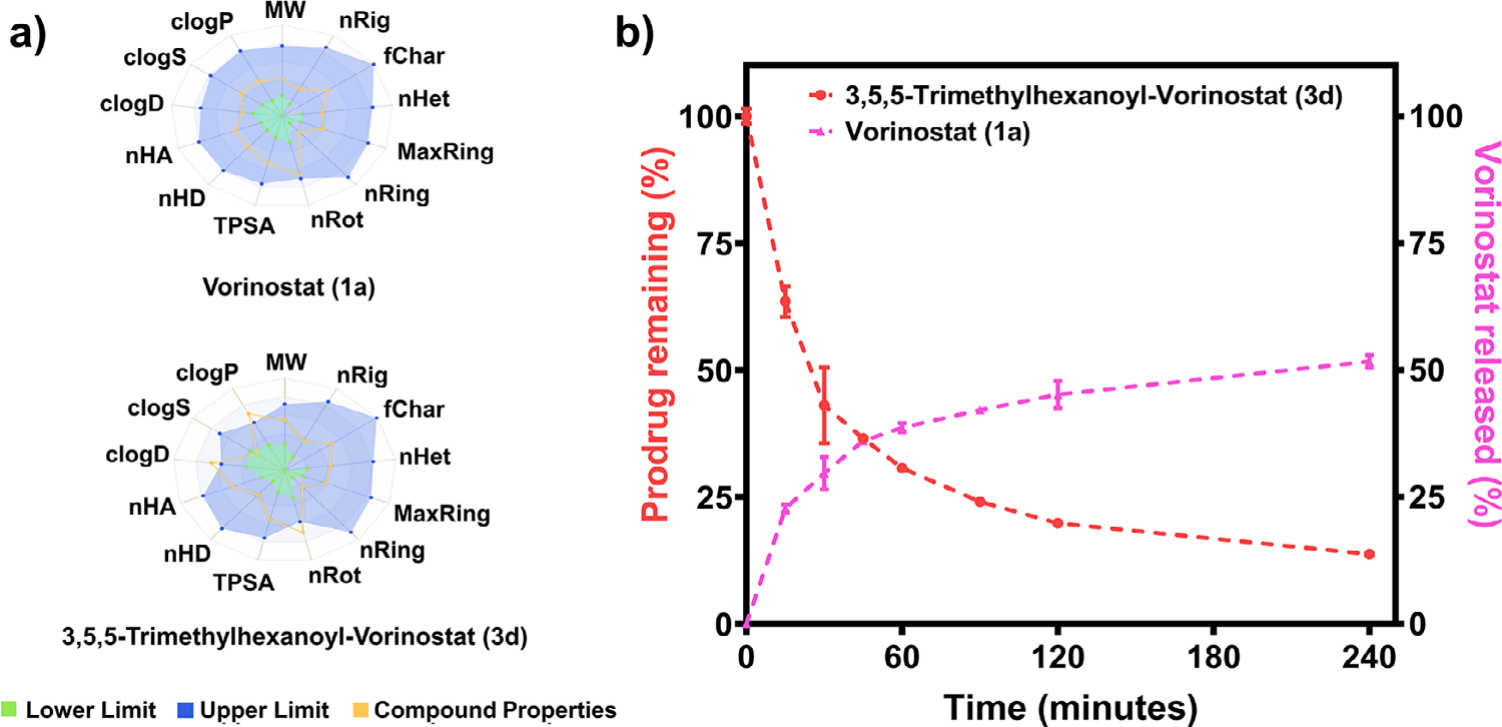
In silico ADMET profile and in vitro plasma stability of prodrug 3d. (a) Bioradar representation of predicted ADMET properties of vorinostat (1a) and 3,5,5‐Trimethylhexanoyl‐Vorinostat (3d). (b) In vitro plasma stability profile of prodrug 3d (3,5,5‐Trimethylhexanoyl‐Vorinostat), demonstrating hydrolytic reduction over time, to release active vorinostat.

The in silico ADMET analysis was performed to predict pharmacokinetic profile and drug‐likeness properties of the synthesized lipid‐vorinostat conjugates (3a‐3d) in comparison to the native vorinostat (1a) (Figures 5a and S18; Tables 3 and S1) [27]. Interestingly, the synthesized potent conjugate 3d demonstrated favorable physicochemical properties consistent with drug‐likeness criteria. It possesses six hydrogen bond acceptors and two hydrogen bond donors within the optimal range for drug‐like molecules, with molecular weights between 100–500 g/mol. The computed cLog P for 3d was 4.17, remarkably higher than 1a (cLog P = 1.177), indicating improved lipophilicity due to conjugation with 3,5,5‐Trimethylhexanoyl chloride (2d). While the clog S value of > –5 suggests acceptable aqueous solubility, shows a relative decrease compared to 1a, representing a common trade off associated with lipid‐based conjugation strategies [28]. Additionally, 3d exhibited higher topological polar surface area (TPSA) of 84.5 Å^2^ and an increased number of rotatable bonds compared to 1a, as an attribute to lipid‐linked structure. These values collectively indicate that compound 3d complies with Lipinski's Rule of Five.

**TABLE 3:** Predicted ADMET profile of vorinostat (1a) and 3,5,5‐Trimethylhexanoyl‐Vorinostat (3d).

Parameters	1a	3d
no. of hydrogen bond acceptor (nHA)	5	6
no. of hydrogen bond donor (nHD)	3	2
Topological surface area (TPSA)	78.43	84.5
no. of Rotatable bond (nRot)	10	16
Quantitative Estimate of Drug‐likeness (QED)	0.383	0.401
Synthetic accessibility score (Synth)	1.695	2.819
Medicinal chemistry evolution‐18 (MCE‐18)	6	22
Lipinski Rule	0	0
Pfizer Rule	0	0
Golden Triangle	0	0
cLogS	−2.19608	−4.93557
cLogD	1.381956	3.7785
cLogP	1.176501	4.169787
caco2 Permeability	−5.17816	−4.97162
MDCK Permeability	−4.04756	−4.66755
PAMPA	0.967456	0.950008
P‐glycoprotein (Pgp) inhibitor	0.001384	0.000138
P‐glycoprotein (Pgp)substrate	0.522878	0.003858
OATP1B1 inhibitor	0.001352	0.001013
OATP1B3 inhibitor	0.196893	0.697608
Breast Cancer Resistance Protein (BCRP) inhibitor	1.95E‐05	7.53E‐09
Bile Salt Export Pump (BSEP)	0.500616	0.229578
Blood brain barrier (BBB) Penetration	0.871127	0.993878
Multidrug Resistance‐associated Protein‐1 (MRP1) inhibitor	0.871347	0.837673
Plasma Protein Binding (PPB)	77.94458	94.45631

cLogS: logarithm of aqueous solubility cLogD: logarithm of n‐octanol/water distribution coefficient cLogP: logarithm of the n‐octanol/water distribution coefficient at pH = 7.4; caco2: Cancer coli‐2; MDCK: Madin‐Darby Canine Kidney; PAMPA: Parallel Artificial Membrane Permeability Assay; OATP: Organic Anion Transporting Polypeptide.

The predicted pharmacokinetic profiling also supported drug‐likeness, *viz*. Caco‐2 permeability improved from ‐5.17 (1a) to ‐4.97 (3d), and MDCK permeability slightly decreased from ‐4.40 (1a) to ‐4.66 (3d). PAMPA values remained comparable (0.96 for 1a and 0.95 for 3d), the lipid modification may offer distinct advantages in cellular retention. Further, the computational model predicts that 3d is a significantly poorer substrate for P‐glycoprotein (Pgp) and BCRP efflux transporters as compared to vorinostat (1a). This substantial reduction in predicted efflux affinity suggests that lipid conjugation may enhance net intracellular accumulation by bypassing active transport‐mediated resistance. The plasma protein binding (PPB) increased from 77.94% (1a) to 94.45% (3d), and blood‐brain barrier permeability improved from 0.87 to 0.994. Overall, LDC increases lipophilicity, the predicted ADMET profile suggests that compound **3d** retains favorable drug‐like attributes while potentially gaining an advantage through reduced susceptibility to cellular efflux mechanisms.

### 2.8 In Vitro Plasma Stability of Prodrug 3d

A prodrug, being pharmacologically inactive, must undergo bioconversion to release the parent drug in order to exert its intended therapeutic effect [16, 17]. Therefore, in vitro plasma stability study of compound 3d was performed to evaluate its susceptibility to enzymatic hydrolysis and its ability to release active vorinostat, under physiological conditions. Upon incubation in plasma, prodrug 3d demonstrated a clear time‐dependent decrease in concentration, resulting in the release of active vorinostat, indicating efficient bioconversion (Figure 5b) [29]. The observed conversion was consistent with prodrug behavior designed to balance plasma stability with efficient release of the active compound [14, 30, 31]. Further, the prodrug demonstrated a estimated hydrolysis rate constant of 0.0074 per minute and a plasma half‐life (t_1/2_) of ∼94 minutes. Although the conversion of **3d** to **1a** confirms the feasibility of the prodrug design, the combined percentage of **3d** and **1a** at 240 min accounted for ∼65 % of the total, indicating that the remaining likely consisted of additional hydrolysis products that were not identified. Comprehensive identification and characterization of these metabolites will require LC‐MS/MS‐based metabolite profiling, which was beyond the scope of the present study.

## 3 Conclusion

In this study, lipid‐conjugates of vorinostat were successfully synthesized through acylation with short‐chain fatty acid derivatives, yielding a prodrug with improved lipophilicity. The selected fatty acid acyl chlorides were chosen to preliminarily evaluate the influence of chain length and lipophilicity on the biological and physicochemical properties of the corresponding prodrugs. This diverse set provides a foundational library for early‐stage structure‐activity relationship (SAR) investigations while primarily serving to demonstrate the feasibility of the prodrug strategy and identify promising candidates, rather than to establish a comprehensive SAR. Among the synthesized conjugates, compound 3d, functionalized with 3,5,5‐Trimethylhexanoyl group, showed significantly greater cytotoxicity against the A549 and 4T1 cancer cell lines than native vorinostat. This enhanced activity was supported by increased caspase‐3/7 activity, ROS generation, MMP loss, and inhibition of cell proliferation, collectively indicating stronger apoptosis induction. In silico ADMET predictions suggested that compound 3d meets drug‐likeness criteria and exhibits improved predicted permeability and plasma protein binding in comparison to vorinostat; however, these predictions require further experimental validation. Moreover, an in vitro plasma stability study showed that prodrug 3d undergoes enzymatic hydrolysis, releasing the active vorinostat, supporting the proposed prodrug approach. The synthesized compound 3d shows promise for further preclinical development. Future studies should focus on the experimental validation of the predicted ADMET properties through in vitro assays, as well as in vivo pharmacokinetic and pharmacodynamic studies, to comprehensively assess the therapeutic potential of compound 3d.

## 4 Materials and Methods

### 4.1 Chemicals and Reagents

Vorinostat (Mw: 264.32 g/mol; CAS No.: 149647‐78‐9) was procured from BLD Pharmatech Pvt Ltd (Telangana, India). The acid chloride derivatives of fatty acids, Isovaleryl chloride (Mw: 120.58 g/mol; CAS No.: 108‐12‐3), Nonanoyl chloride (Mw: 176.68 g/mol; CAS No.: 764‐85‐2), Heptanoyl chloride (Mw: 148.63 g/mol; CAS No.: 2528‐61‐2), and 3,5,5‐Trimethylhexanoyl chloride (Mw: 176.68 g/mol; CAS No.: 36727‐29‐4), were procured from Tokyo Chemical Industries (Japan, Tokyo). Human plasma was purchased from Sigma‐Aldrich. All the reagents were of analytical grade and utilized without further purification unless otherwise stated.

### 4.2 Synthesis and Characterization of Vorinostat Prodrug

#### 4.2.1 General Procedure

Vorinostat (**1a**, 0.756 mmol, 1 equiv.) was dissolved completely in pyridine and stirred at room temperature [32]. To this, respective fatty acid chloride (0.907 mmol, 1.2 equiv.) either Isovaleryl chloride (2a), Nonanoyl chloride (2b), Heptanoyl chloride (2c), or 3,5,5‐Trimethylhexanoyl chloride (2d) was added dropwise. The reaction mixture was continuously stirred in a sealed vessel at room temperature for 36 hours. Reaction was monitored by thin‐layer chromatography (TLC) with UV visualization. Upon completion of the reaction, pyridine was removed using a rotary evaporator (IKA RV10), and the crude product was purified by column chromatography employing silica as stationary phase. The purified lipid‐vorinostat conjugates (3a–3d) were characterized by nuclear magnetic resonance spectroscopy (NMR), high‐resolution mass spectrometry (HRMS) and high‐performance liquid chromatography (HPLC) (C‐18 column: 250 × 4.6 mm, 5 µm, methanol: 0.1% TFA‐water (80:20 v/v), flow: 0.8 ml/min). The synthesized compounds were stored at −20 °C in amber vials under an inert atmosphere to prevent degradation.

#### 4.2.2 NMR and Spectral Characterization

**Vorinostat (1a)**: N1‐hydroxy‐N8‐phenyloctanediamide. white solid. **^1^H NMR (500 MHz, DMSO‐d_6_)** δ10.34 (OH), 9.84(s, 1H), 8.67 (s, 1H), 7.58(d, J = 5 Hz, 2H), 7.27(t, J = 10 Hz, 2H), 7.01 (t, J = 5 Hz, 1H), 7.28 (t, J = 7.5 Hz, 2H), 1.94 (t, J = 7.5 Hz, 2H) 1.60‐1.54 (m, 2H), 1.51‐1.46(m, 2H) 1.27(t, J = 3.5 Hz, 4 H); **^13^C NMR (125 MHz, DMSO‐d_6_)** δ 171.3, 169.2, 139.36, 128.67, 122.97, 119.09, 36.41, 32.29, 28.46, 25.08; **HRMS (ESI) (m/z)**: cal. For C_14_H_21_N_2_O_3_^+^: 265.1547; found: 265.1552

**Isovaleryl‐Vorinostat (3a)**: N1‐((3‐methylbutanoyl)oxy)‐N8‐phenyloctanediamide. white solid (53%). **^1^H NMR (500 MHz, DMSO‐d_6_)** δ 11.53 (s, 1H), 9.84 (s, 1H), 7.58 (d, J = 5 Hz, 2H), 7.27 (t, J = 7.5 Hz, 2H), 7.02(t, J = 7.5, 1H), 2.31‐2.27(m, 4H), 2.10(t, J = 7.5, 2H), 2.04‐1.98 (m, 1H), 1.59‐1.51(m, 4H), 1.30 (s, 4H), 0.95 (d, J = 5, 6H); **^13^C NMR (125 MHz, DMSO‐d_6_)** δ 171.26, 170.48, 170.16, 139.60, 128.88, 123.17, 119.27, 36.38, 31.88, 28.37, 28.23, 25.40, 24.98, 24.65, 21.97; **HRMS (ESI) (m/z)**: cal. For C_19_H_29_N_2_O_4_^+^: 349.2122; found: 349.2123

**Nonanoyl‐Vorinostat (3b)**: N1‐(nonanoyloxy)‐N8‐phenyloctanediamide. white solid (65%) **^1^H NMR (500 MHz, DMSO‐d_6_)** δ 11.53(s, 1H), 9.84 (s, 1H), 7.58 (d, J = 5.0 Hz, 2H), 7.27 (t, J = 10 Hz, 2H), 7.01 (t, J = 7.5 Hz, 1H), 2.41 (t, J = 7.5 Hz, 2H), 2.28 (t, J = 7.5 Hz, 2H), 2.10 (t, J = 7.5 Hz, 2H), 1.57‐1.51 (m, 6H), 1.29 (d, J = 25 Hz, 14H), 0.85 (t, J = 5 Hz, 3H); **^13^C NMR (125 MHz, DMSO‐d_6_)** δ 171.26, 169.84, 139.35, 128.65, 122.95, 119.07, 36.39, 31.90, 31.23, 30.96, 28.60, 28.55, 28.40, 28.28, 28.26, 25.03, 24.70, 24.39, 22.10, 13.98; **HRMS (ESI) (m/z)**: Cal. For C_23_H_37_N_2_O_4_^+^: 405.2748; found: 405.2750.

**Heptanoyl‐Vorinostat (3c)**: N1‐(heptanoyloxy)‐N8‐phenyloctanediamide. white solid (58%) **^1^H NMR (500 MHz, DMSO‐d_6_)** δ 11.53 (s, 1H), 9.84(s, 1H), 7.59 (d, J = 10.0 Hz, 2H), 7.27 (t, J = 10 Hz, 2H), 7.01 (t, J = 7.5 Hz, 1H), 2.41 (t, J = 7.5 Hz, 2H), 2.28 (t, J = 7.5 Hz, 2H), 2.10 (t, J = 7.5 Hz, 2H), 1.57‐1.51 (m, 6H), 1.28 (m, 10H), 0.85 (t, J = 7.5 Hz, 3H); **^13^C NMR (125 MHz, DMSO‐d_6_)** δ 171.25, 169.83, 139.34, 128.64, 122.94, 119.06, 36.39, 31.90, 30.95, 30.84, 28.39, 28.25, 27.93, 25.02, 24.70, 24.35, 21.95, 13.89; **HRMS (ESI) (m/z)**: Calc. for C_21_H_33_N_2_O_4_^+^; 377.2435; found: 377.2439

**3,5,5‐Trimethylhexanoyl‐Vorinostat (3d)**: N1‐phenyl‐N8‐((3,5,5‐trimethylhexanoyl)oxy)octanediamide. white solid (57%). **^1^H NMR (500 MHz, DMSO‐d_6_)** δ 11.52 (s, 1H), 9.84 (s, 1H), 7.58 (d, J = 5.0 Hz, 2H), 7.27 (t, J = 10 Hz, 2H), 7.01 (t, J = 7.5 Hz, 1H), 2.42‐2.37 (m, 1H), 2.30‐2.23 (m, 3H),), 2.10 (t, J = 7.5 Hz, 2H), 1.99‐1.93 (m,1H), 1.59‐1.51(m, 4H), 1.31‐1.27 (m, 5H), (1.12‐1.08 (m, 1H), 0.98 (d, J = 5.0 Hz, 3H), 0.88 (s, 9H); **^13^C NMR (125 MHz, DMSO‐d_6_)** δ 171.25, 170.45, 169.81, 139.34, 128.65, 122.95, 119.06, 49.75, 40.51, 36.39, 31.90, 30.76, 29.79, 28.40, 28.26, 26.71, 25.02, 24.70, 22.17; **HRMS (ESI) (m/z)**: Calc. for C_23_H_37_N_2_O_4_^+^: 405.2748; found: 405.2749

### 4.3 Cell Culture

Adenocarcinoma human alveolar basal epithelial cells (A549, RRID:CVCL_0023), murine macrophage cells (RAW 264.7, RRID:CVCL_0493), and murine fibroblast cells (L929, RRID:CVCL_0462) were procured from the National Centre for Cell Science (NCCS, Pune, India) and maintained in Dulbecco's Modified Eagle Medium (DMEM, Gibco). Murine mammary carcinoma cells (4T1, RRID:CVCL_0125) were sourced from the American Type Culture Collection (ATCC, USA) and cultured in Roswell Park Memorial Institute medium (RPMI, Gibco). All culture media were supplemented with 10% (v/v) Fetal bovine serum (MP Biomedicals) and 1% (v/v) antibiotic‐antimycotic solution (Gibco). Cell lines were cultured in a humidified incubator at 37 °C with 5% CO_2_ and routinely passaged for continued use.

#### 4.3.1 Cytotoxicity Assay

The cytotoxic effects of lipid‐vorinostat conjugates were evaluated against A549 and 4T1 cell lines using the CCK‐8 assay at 48 hours [33, 34]. For each cell line, 5 × 10^3^ cells per well were seeded into 96‐well plates. After cells attained desired confluency, the treatment was performed with varying concentrations (0.5, 1, 5, 10, and 20 µM) of vorinostat (1a) and its conjugates (3a‐d). After 48 hours of treatment, the medium was replaced with 10% CCK‐8 solution in DMEM and incubated for 3 hours at 37°C with 5% CO_2_. The absorbance was recorded using a microplate reader (Synergy‐H1, BioTek275) at 450 nm. The following equation was used to determine cell viability:

(1) $$\mathrm{Cell}\;\mathrm{viability}\;\left(\%\right)=\frac{OD_{\mathrm{test}}}{OD_{\mathrm{control}}}\;\times 100$$

The IC_50_ value was determined using GraphPad Prism 10.4.0 (GraphPad Software Inc., CA, USA) by fitting the dose‐response data to a nonlinear second order polynomial (quadratic) regression model. The regression equation was used to interpolate the concentration corresponding to 50% inhibition. The same regression approach was consistently applied to all tested compounds to enable comparative evaluation of their cytotoxic activities under identical experimental conditions. Additionally, biocompatibility of the lead analogue (3d) and vorinostat (1a) was assessed on normal cell lines RAW 264.7 and L929, and cell viability was assessed similarly using the CCK‐8 assay.

#### 4.3.2 Caspase 3/7 Activity Assay

For evaluation of caspase‐3/7 activity, 5 × 10^4^ A549 and 4T1 cells were seeded into 12‐well plates and treated with two different concentrations (5 and 10 µM) of compounds 1a and 3d for 24 hours [34, 35]. Apoptotic activity was measured using the Caspase‐3/7 Green Detection Reagent (CellEvent, Invitrogen, Thermo Fisher Scientific). The reagent was diluted 1:400 in 2 mL of Phosphate buffer saline (PBS), and 5 µL of this diluted solution was added to each well. After incubation for 30 minutes at 37°C, GFP fluorescence was quantified using an automated cell counter (Countess 3FL, Invitrogen, Thermo Fisher Scientific, Singapore) with excitation and emission at 502 nm and 530 nm, respectively.

#### 4.3.3 ROS Assay

To assess the intracellular ROS, a cell permeable fluorogenic dye 2′,7′‐dichlorofluorescin diacetate (DCFDA) was used [36, 37]. In 12‐well plates, A549 and 4T1 cells (5 × 10^4^ cells per well) were seeded and allowed to grow for 24 hours. Subsequently, cells were treated with 5 and 10 µM concentrations of compounds 1a and 3d for 24 hours. Afterwards, cells were washed with PBS and incubated with (400 µL of 5 µM) DCFDA for 30 minutes in the dark. Following incubation, the dye was removed, cells were washed twice with PBS, and ROS‐induced fluorescence was visualized using a fluorescence microscope. Quantitative measurement of ROS was performed using a microplate spectrophotometer (Synergy‐H1, BioTek 275) by recording fluorescence intensity with excitation at 498 nm and emission at 522 nm.

#### 4.3.4 Mitochondrial Membrane Potential Loss Analysis

The effect of compounds 1a and 3d on MMP was assessed using the JC‐1 dye (Tetraethylbenzimidazolylcarbocyanine iodide, Invitrogen, Thermo Fisher Scientific) [14, 38]. A549 and 4T1 cells (5 × 10^4^ cells per well) were seeded in 12‐well plates and allowed to attain confluency for 24 hours. Cells were then treated with 5 and 10 µM of 1a and 3d for 24 hours. After treatment, cells were washed with PBS and incubated with 2 µM JC‐1 dye for 20 minutes at 37°C in the dark. Fluorescent images were captured using a fluorescent cell imager (ZOETM, Bio‐Rad), and fluorescence intensity was quantified with ImageJ software to evaluate changes in MMP.

#### 4.3.5 Scratch Wound Assay

Cells (5×10^4^ per well) were seeded in 6‐well plates and cultured to obtain confluent monolayer [39, 40]. To create wound, a linear scratch was made at the center of each well. After scratching, wells were washed with PBS and fresh medium containing compounds **1a** and **3d** at concentrations of 5 and 10 µM was added. Wound closure was monitored by capturing images at 0, 24, and 48 hours to assess cell migration and wound healing. The wound closure percentage at 24 and 48 hours was determined using ImageJ software, relative to the initial scratch area measured at 0 hour.

### 4.4 ADMET Profile of Vorinostat Conjugates

The ADMET (Absorption, Distribution, Metabolism, Excretion, and Toxicity) and pharmacokinetic profiles of vorinostat (1a) and its lipid‐vorinostat conjugates (3a–3d) were predicted using the online web server ADMETlab 3.0 (https://admetlab3.scbdd.com/server/evaluation) [41].

### 4.5 In Vitro Plasma Stability of Prodrug 3d

The in vitro plasma stability of the potent vorinostat conjugate, 3,5,5‐Trimethylhexanoyl‐Vorinostat‐ (**3d**), was performed in human plasma [29]. The assay was performed with the addition of the sample to 2 mL of preheated plasma solution yielding a concentration of 200 µM. The assay was performed with continuous shaking at 37°C in a Matrix orbital shaker (IKA, Germany). A 100 µL of sample was drawn at intervals of 0, 15, 30, 45, 60, 90, 120, and 240 minutes. Subsequently, 200 µL of ACN was added to deproteinize the plasma. The obtained samples were vortexed for 1 min and then centrifuged for 15 minutes at 4°C at 14000 rpm. The obtained supernatant was quantified for the extent of 3d and 1a using HPLC. The in vitro plasma half‐life (t_1/2_) was determined using the following equation:

(2) $$t_{1/2}=\frac{0.693}{k}\;$$

where k: hydrolysis rate constant determined from the slope of the linear regression fit of the natural logarithm of the prodrug remaining vs time [29].

### 4.6 Statistical Analysis

All statistical analyses for in vitro cell culture assays were performed using GraphPad Prism 10.4.0 (GraphPad Software Inc., CA, USA). Data are expressed as mean ± standard deviation (SD). The results were analyzed using one‐way analysis of variance (one‐way ANOVA), followed by Tukey's post hoc test for multiple comparison. The p‐value < 0.05 was considered statistically significant.

## Author Contributions

**N.T**.: investigation, methodology, analysis, writing – original draft; **N.B**.: investigation, methodology, analysis, writing – original draft; **S.K.Y**.: writing – review and editing, and formal analysis; **A.S**.: formal analysis, supervision, writing – review and editing, funding acquisition, supervision, and conceptualization.

## Funding

A.S. and N.T. acknowledge the support obtained from CSIR (MLP‐204) and ICMR New Delhi (5/9/1423/2022‐Nut).

## Conflicts of Interest

The authors declare no conflicts of interest.

## Supporting information



The Supporting Information contains HRMS, HPLC, NMR, graph for cytotoxicity assay, biocompatibility assay, and predicted ADMET profile.
**Supporting File 1**: cbdv71726‐sup‐0001‐SuppMat.docx.

## Data Availability

The data supporting the findings of this study are included within the article and its Supporting Information.
