# Supplementary material for: Synthesis and Biological Evaluation of a Lipophilic Vorinostat Prodrug
Source: Chem Biodivers. 2026 Sep 27;23(9):e71726. doi: 10.1002/cbdv.71726 (PMC13616466; doi:10.1002/cbdv.71726)
Supplement: Supplementary file 1 [file CBDV-23-e71726-s001.docx]

**Supplementary Information**

**Synthesis and Biological Evaluation of a Lipophilic Vorinostat Prodrug**

Navneet Thakur^1,2#^, Neha Bhardwaj^1,2#^, Sudesh Kumar Yadav^2,3^, Ankit Saneja^1,2^*

^1^Formulation Laboratory, Dietetics and Nutrition Technology Division, CSIR-Institute of Himalayan Bioresource Technology, Palampur-176061, Himachal Pradesh, India

^2^Academy of Scientific and Innovative Research (AcSIR), Ghaziabad-201002, India

^3^CSIR-Institute of Himalayan Bioresource Technology, Palampur-176061, Himachal Pradesh, India

^#^Co-first authors

***Corresponding Author**

Dr. Ankit Saneja

Senior Scientist

Dietetics and Nutrition Technology Division,

CSIR – Institute of Himalayan Bioresource Technology,

Palampur, 176061

Himachal Pradesh, India

E-mail: [ankitsaneja@ihbt.res.in](mailto:ankitsaneja@ihbt.res.in); [ankitsaneja.ihbt@gmail.com](mailto:ankitsaneja.ihbt@gmail.com)

Tel: 91-1894-233339, Ext: 485

| Table of content | | |
| --- | --- | --- |
| S no. | Figure | |
| 1 | Figure S1 | HRMS spectra of vorinostat (**1a**) |
| 2 | Figure S2 | HPLC chromatogram of vorinostat (**1a**) |
| 3 | Figure S3 | ^1^H & ^13^C NMR spectra of vorinostat (**1a**) in DMSO-d_6_ |
| 4 | Figure S4 | HRMS spectra of Isovaleryl-Vorinostat (**3a**) |
| 5 | Figure S5 | HPLC chromatogram of Isovaleryl-Vorinostat (**3a**) |
| 6 | Figure S6 | ^1^H & ^13^C NMR spectra of Isovaleryl-Vorinostat (**3a**) in DMSO-d_6_ |
| 7 | Figure S7 | HRMS spectra of Nonanoyl-Vorinostat (**3b**) |
| 8 | Figure S8 | HPLC chromatogram of Nonanoyl-Vorinostat (**3b**) |
| 9 | Figure S9 | ^1^H & ^13^C NMR spectra of Nonanoyl-Vorinostat (**3b**) in DMSO-d_6_ |
| 10 | Figure S10 | HRMS spectra of Heptanoyl-Vorinostat (**3c**) |
| 11 | Figure S11 | HPLC chromatogram of Heptanoyl-Vorinostat (**3c**) |
| 12 | Figure S12 | ^1^H & ^13^C NMR spectra of Heptanoyl-Vorinostat (**3c**) in DMSO-d_6_ |
| 13 | Figure S13 | HRMS spectra of 3,5,5-Trimethylhexanoyl-Vorinostat (**3d**) |
| 14 | Figure S14 | HPLC chromatogram of 3,5,5-Trimethylhexanoyl-Vorinostat (**3d**) |
| 15 | Figure S15 | ^1^H & ^13^C NMR spectra of 3,5,5-Trimethylhexanoyl-Vorinostat (**3d**) in DMSO-d_6_ |
| 16 | Figure S16 | Cytotoxicity assay: Cell viability graph of Vorinostat(**1a**) and 3,5,5-Trimethylhexanoyl-Vorinostat (**3d**) against: (**a**) A549, and (**b**) 4T1 cells, at different concentration *viz*. 0.5, 1, 5, 10 and 20 µM. |
| 17 | Figure S17 | Biocompatibility assay of Vorinostat (**1a**) and vorinostat-3,5,5-trimethylhexanoyl (**3d**) on RAW 264.7 and L929 |
| 18 | Figure S18 | Bioradar representation of predicted ADMET properties of vorinostat conjugates: Isovaleryl-vorinostat (3a), Nonanoyl-vorinostat (3b), and Heptanoyl-Vorinostat (3c). |
| **Table** | | |
| 1 | Table S1 | ADMET profile of Isovaleryl-Vorinostat (3a), Nonanoyl-Vorinostat (3b) and Heptanoyl-Vorinostat (3c). |

**Figure S1**. HRMS spectra of vorinostat (**1a**) representing the [M+H]^+^ value 265.1552.

**Figure S2.** HPLC chromatogram of vorinostat (**1a**) representing RT at 3.86 minutes.

**a)**

**b)**

**Figure S3.** NMR spectra of vorinostat (**1a**) in DMSO-d_6_: **a**) ^1^H NMR spectra (500 MHz) and **b**) ^13^C NMR spectra (125 MHz).

**Figure S4.** HRMS spectra of Isovaleryl-Vorinostat (**3a**) representing the [M+H]+ value 349.2123.

**Figure S5**. HPLC chromatogram of Isovaleryl-Vorinostat (**3a**) representing RT at 4.90 minutes

**a)**

**b)**

**Figure S6**. NMR spectra of Isovaleryl-Vorinostat (**3a**) in DMSO-d_6_: a) ^1^H NMR spectra (500 MHz) and b) ^13^C NMR spectra (125 MHz).

**Figure S7**. HRMS spectra of Nonanoyl-Vorinostat (**3b**) representing the [M+H]+ value 405.2750.

**Figure S8**. HRMS spectra of Nonanoyl-Vorinostat (**3b**) representing RT at 10.04 minutes.

**b)**

**a)**

**Figure S9.** NMR spectra of Nonanoyl-Vorinostat (**3b**) in DMSO-d_6_: a) ^1^H NMR spectra (500 MHz) and b) ^13^C NMR spectra (125 MHz).

**Figure S10.** HRMS spectra of Heptanoyl-Vorinostat (**3c**) representing the [M+H]+ value 377.2439.

**Figure S11.** HPLC chromatogram of Heptanoyl-Vorinostat (**3c**) representing RT at 6.54 minutes.

**b)**

**a)**

**Figure S12.** NMR spectra of Heptanoyl-Vorinostat (**3c**) in DMSO-d_6_: **a**) ^1^H NMR spectra (500 MHz) and **b**) ^13^C NMR spectra (125 MHz)

**Figure S13.** HRMS spectra of 3,5,5-Trimethylhexanoyl-Vorinostat (**3d**) representing the [M+H]^+^ value 405.2749.

**Figure S14**. HPLC chromatogram of 3,5,5-Trimethylhexanoyl-Vorinostat (**3d**) representing RT at 8.21 minutes.

**a)**

**b)**

**Figure S15**. NMR spectra of 3,5,5-Trimethylhexanoyl-Vorinostat (**3d**) in DMSO-d_6_: **a**) ^1^H NMR spectra (500 MHz) and b) ^13^C NMR spectra (125 MHz).

**Figure S16**. Cytotoxicity assay: Cell viability graph of Vorinostat(**1a**) and 3,5,5-Trimethylhexanoyl-Vorinostat (**3d**) against: (**a**) A549, and (**b**) 4T1 cells, at different concentration *viz*. 0.5, 1, 5, 10 and 20 µM.

**Figure S17**. Biocompatibility assay of the vorinostat (**1a**) and 3,5,5-Trimethylhexanoyl-Vorinostat (**3d**) in normal cell line: (**a**) RAW 264.7 and (**b**) L929 cells.

**Figure S18.** Bioradar representation of predicted ADMET properties of vorinostat conjugates: Isovaleryl-vorinostat (3a), Nonanoyl-vorinostat (3b), and Heptanoyl-Vorinostat (3c).

**Table S1.** ADMET profile of Isovaleryl-Vorinostat (3a), Nonanoyl-Vorinostat (3b) and Heptanoyl-Vorinostat (3c).

| Parameters | 3a | 3b | 3c |
| --- | --- | --- | --- |
| no. of hydrogen bond acceptor (nHA) | 6 | 6 | 6 |
| no. of hydrogen bond donor (nHD) | 2 | 2 | 2 |
| Topological surface area (TPSA) | 84.5 | 84.5 | 84.5 |
| no. of Rotatable bond (nRot) | 14 | 19 | 17 |
| Quantitative Estimate of Drug-likeness (QED) | 0.5 | 0.307 | 0.392 |
| Synthetic accessibility score (Synth) | 2.15 | 2.15 | 2.099 |
| Medicinal chemistry evolution-18 (MCE-18) | 8 | 7 | 7 |
| Lipinski Rule | 0 | 0 | 0 |
| Pfizer Rule | 0 | 0 | 0 |
| Golden Triangle | 0 | 0 | 0 |
| cLogS | -4.06726 | -4.82624 | -4.59955 |
| cLogD | 2.951724 | 3.869918 | 3.524634 |
| cLogP | 2.725153 | 4.581108 | 3.797266 |
| caco2 Permeability | -5.02364 | -5.15167 | -5.13822 |
| MDCK Permeability | -4.45647 | -4.74562 | -4.6838 |
| PAMPA | 0.970656 | 0.6887 | 0.875573 |
| P-glycoprotein (Pgp) inhibitor | 4.95E-05 | 0.00039 | 0.001109 |
| P-glycoprotein (Pgp)substrate | 0.002066 | 0.002866 | 0.005559 |
| OATP1B1 inhibitor | 0.005769 | 0.00298 | 0.001441 |
| OATP1B3 inhibitor | 0.766203 | 0.993822 | 0.875782 |
| Breast Cancer Resistance Protein (BCRP) inhibitor | 2.63E-08 | 5.31E-07 | 3.00E-07 |
| Bile Salt Export Pump (BSEP) | 0.819103 | 0.828954 | 0.886639 |
| Blood brain barrier (BBB) Penetration | 0.974263 | 0.573686 | 0.806076 |
| Multidrug Resistance-associated Protein-1 (MRP1) inhibitor | 0.950503 | 0.980782 | 0.975167 |
| Plasma Protein Binding (PPB) | 85.86226 | 96.92873 | 95.4931 |

cLogS: logarithm of aqueous solubility	cLogD: logarithm of n-octanol/water distribution coefficient	cLogP: logarithm of the n-octanol/water distribution coefficient at pH=7.4; caco2: Cancer coli-2; MDCK: Madin-Darby Canine Kidney; PAMPA: Parallel Artificial Membrane Permeability Assay; OATP: Organic Anion Transporting Polypeptide.
